# Immune cells play a critical role in cytokine- and endotoxin-mediated endothelial permeability

**DOI:** 10.1371/journal.pone.0329700

**Published:** 2025-08-14

**Authors:** Patricia Gogesch, Samira Ortega Iannazzo, Nicole Rupp, Joachim Rom, Markus Kreuz, Kristin Reiche, Martina Anzaghe, Zoe Waibler, on behalf of the imSAVAR Consortium

**Affiliations:** 1 Division of Immunology, Paul-Ehrlich-Institut, Langen, Germany; 2 Department of Gynecology, Varisano Klinikum sFrankfurt Höchst, Frankfurt, Germany; 3 Department of Diagnostics, Fraunhofer Institute for Cell Therapy and Immunology IZI, Leipzig, Germany; Eötvös Loránd Research Network Biological Research Centre, HUNGARY

## Abstract

Vascular leakage (VL) is a severe pathology occurring in a broad range of scenarios, e.g., during sepsis, cytokine storms, or as side effect of immunotherapies. Its severity is underlined by the high lethality rate of 20−30% for the systemic capillary leakage syndrome. While many compounds are reported to affect endothelial cell (EC)-activation, exact mechanisms behind VL remain unclear. We analyzed activation, viability, cytokine secretion, and relative permeability of human umbilical vein endothelial cells (HUVECs) upon treatment with 16 different stimuli. Relative HUVEC-permeability was assessed in a trans-well-based leakage assay in presence or absence of human peripheral blood mononuclear cells (PBMCs). HUVEC-activation is characterized by correlating upregulation of intercellular adhesion molecule (ICAM)-1, vascular cell adhesion molecule (VCAM)-1, and E-selectin, as well as production of interleukin (IL)-8, monocyte chemoattractant protein (MCP)-1, and IL-6. Strong HUVEC-activation and reduced viability was observed upon treatment with IL-1β, tumor necrosis factor (TNF)-α, a TGN1412-induced cytokine cocktail (SN_TGN1412_), thrombin, and lipopolysaccharide (LPS). Only thrombin, SN_TGN1412,_ and vascular endothelial growth factor (VEGF) led to increased relative permeability, while other compounds associated with vascular leakage, including TNF-α, IL-1β, or LPS, had no direct effect on relative HUVEC-permeability. Interestingly, co-cultures with PBMCs mediated IL-1β- and LPS- but not TNF-α-induced relative HUVEC-permeability. In our study, we show that HUVEC activation upon direct stimulation does not necessarily result in increased relative permeability or massive cytokine production. Interestingly, we could demonstrate that activated HUVECs respond with a conserved pattern of markers, independent from the stimulus used. Moreover, we observed that the interplay with immune cells is critical to enhance relative HUVEC-permeability, which however depended on the stimulus applied suggesting different mechanisms of immune-mediated VL. A better understanding of VL will uncover potential treatment-targets for patients suffering from VL and help to improve safety-assessment of leakage-associated immunotherapies.

## Introduction

Pathologically increased endothelial permeability can culminate in vascular leakage (VL), a serious condition observed in several contexts, e.g., as secondary disease during sepsis, infections, or immunotherapies, but also as idiopathic disease, thus with unknown source of induction. Unspecific clinical manifestations followed by a rapid progression to a massive leak phase lead to underestimation, misdiagnosis, or diagnostic delay of VL. Consequently, VL is associated with poor clinical outcome and can lead to down-stream pathologies such as edema, organ damage, or even multi-organ failure and death, as emphasized by a lethality rate of 20–30% for the capillary leakage syndrome [1,2]. Although VL is a severe condition and subject of research since decades, the mechanisms behind are not completely clarified.

Endothelial cells (ECs) line the vasculature as semi-permeable barrier with its intercellular integrity tightly regulated by laterally expressed surface proteins, including vascular endothelial (VE)-cadherin or junctional adhesion proteins (JAMs) [3,4]. Various compounds, e.g., vasoactive substances, cytokines, or immunological danger signals, can alter the physiological state of ECs [3,5]. Upon activation, ECs can secrete cytokines and chemokines and increase the expression of adhesion molecules such as intercellular adhesion molecule (ICAM)-1, vascular cell adhesion molecule (VCAM)-1, and E-selectin, which mediate the direct interaction with immune cells [6]. For example, ECs stimulated with interleukin (IL)-1β can enhance expression of ICAM-1, VCAM-1, as well as IL-6 and IL-8 secretion [7,8]. However, whether the formation of VL is always a result of EC-activation is not fully understood.

Vasoactive substances, such as thrombin or vascular endothelial growth factor (VEGF) can increase EC-permeability by receptor-ligand-interaction, leading to, e.g., dissociation of VE-cadherin molecules or JAMs, and/or rearrangement of the EC-cytoskeleton [3,9,10]. Moreover, pro-inflammatory cytokines are associated with the formation of VL. For instance, increased permeability of human umbilical vein ECs (HUVECs) induced by tumor necrosis factor (TNF)-α plus interferon (IFN)-γ was further elevated by additional IL-1β-stimulation [11]. The manifestation of a cytokine storm or cytokine release syndrome (CRS), irrespective of its etiology (e.g., infection, sepsis, or during immunotherapy), is often related with the development of VL [2,8,12,13]. In addition to cytokines, also immune cells are reported to promote VL. Accordingly, peripheral blood mononuclear cells (PBMCs) can intensify TNF-α plus IFN-γ-mediated HUVEC-permeability [11]. In turn, depletion of neutrophils was shown to prevent VL-associated lung edema VL [8,14].

Several leakage-associated molecules and their effects on ECs were reported so far. However, studies often focus on one defined stimulus with a restricted set of readouts. A complete characterization taking different groups of stimuli and readouts into account remains to be elucidated. We treated HUVECs with 16 VL-associated compounds including cytokines, vasoactive substances, and immunological danger signals and performed *in vitro* characterization analyses. With a connected dataset, we demonstrated that activated HUVECs respond with a conserved pattern of surfacemarker-expression and cytokine/chemokine-secretion, independent from the type of stimulus used. Nevertheless, HUVEC-activation was not inevitably associated with increased relative permeability. Instead, immune cells were shown to play a critical role in inducing elevated HUVEC-permeability. Interestingly, co-cultivation of HUVECs with PBMCs enhanced relative permeability for most, but not all, immunological stimuli, suggesting different mechanisms for immune-related vascular leakage, which remain to be uncovered.

Besides a broad HUVEC-characterization, in this study, we established an immune-competent *in vitro* EC-permeability-assay and therefore provide a model to unravel mechanisms which contribute to the formation of VL.

## Materials and methods

### Isolation and cultivation of cells

Human umbilical cords from healthy donors were accessed for research purposes on 21/10/2020, 04/03/2021, 31/03/2021, 13/04/2021, 15/06/2021, 06/07/2021, 03/08/2021, 01/09/2021, 28/09/2021, 16/03/2022, and 11/05/2022 (Varisano Klinikum, Frankfurt Höchst, Germany) and HUVECs were isolated as described before [15]. Briefly, surfaces of the umbilical cords were cleaned with 70% ethanol, the vein was rinsed with dPBS, and HUVECs were detached with 0.1% collagenase D-treatment (Roche, Basel, Switzerland) at 37°C for 40 minutes and collected in dPBS. HUVECs were cultivated in cell culture flasks (Greiner, Kremsmünster, Austria) using VascuLife VEGF Endothelial Medium (LifeLine Cell Technology, Frederick, USA) at 37°C with 5% CO_2_ and passaged at a confluence of 80–90% by trypsinization (0.5% trypsin, Sigma-Aldrich, Taufkirchen, Germany). For experiments, HUVECs between passages 2–7 were seeded in EC growth medium (ECGM, PromoCell, Heidelberg, Germany) supplemented with 50 µg/ml gentamycin (Gibco, now Thermo Fisher Scientific, Waltham, USA) and 5 ng/ml amphotericin B (Sigma-Aldrich) in 96-well flat-bottom plates (1x10^6^ cells/ml) or in trans-well inserts (0.4 µm pore size 2x10^5^ cells/ml; both Sarstaedt, Nümbrecht, Germany).

PBMCs were isolated from human buffy coats from healthy donors (Blutspendedienst, Frankfurt am Main, Germany) using pancoll (PAN Biotech, Aidenbach, Germany) and density centrifugation at 460 x g for 20 min. For stimulation, 1x10^6^ PBMCs were seeded in 24-well culture plates (Sarstaedt) and cultivated at 37°C with 5% CO_2_ in X-VIVO^TM^ 15 red (Lonza, Verviers, Belgium). For co-culture experiments, 8x10^4^ pre-stimulated PBMCs were added to 4x10^4^ HUVECs pre-seeded 24 h before in 96-well culture plates and co-cultivated for 24 h.

The confirmation that no ethical approval is needed for the work with Buffy Coats and HUVECs is documented in respective waivers from the ethical committee Frankfurt am Main University (waiver 2023−1272 for Buffy Coats and 22122020 for HUVECs).

### Stimulation of cells

HUVEC-monocultures were treated with the compounds listed in Table 1 for 24 h.

**Table 1:** List of compounds used for stimulation.

Stimuli	Concentration	Supplier
rhIL-1β	1 ng/ml	PeproTech (Cranbury, USA)
rhIL-2	5000 U/ml	Clinigen (London, UK)
rhIL-6	0.1 µg/ml	PeproTech
rhIL-8	3 ng/ml	PeproTech
rhIFN-α	1000 U/ml	Sigma-Aldrich
rhIFN-γ	100 U/ml	PeproTech
rhTNF-α	200 U/ml	PeproTech
SN_TGN1412,_ SN_Ø_	250 µl/ml 250 µl/ml	–
Thrombin (derived from human plasma)	10 U/ml	Sigma-Aldrich
rhVEGF_165_	0.1 µg/ml	PeproTech
Bradykinin	21 µg/ml	Calbiochem (now Sigma-Aldrich)
rhvWF	1 U/ml	Takeda (Berlin, Germany)
LPS (*Salmonella abortus equi)*	100 µg/ml	Sigma-Aldrich
Pam3Cys	1 µg/ml	EMC microcollections (Tübingen,Germany)
Flagellin	10 µg/ml	kindly provided by Stefan Schülke [16]

PBMCs were stimulated with IL1-β, TNF-α, SN_TGN1412,_ or lipopolysaccharide (LPS) using the concentrations given in Table 1 for 5 days or left untreated as control. For generation of the cytokine supernatant (SN), T cells were isolated from PBMCs using the pan T cell isolation kit (Miltenyi Biotec, Bergisch Gladbach, Germany) and were either left untreated or incubated with 1 µg/ml TGN1412 (manufactured by Boehringer Ingelheim, usage was kindly permitted by TheraMAB GmbH) + 2 µg/ml α-IgG (BD Bioscience, San Diego, USA) for 5 days. Cell-free supernatants (unstimulated: SN_Ø_; TGN1412-treated: SN_TGN1412_) from 8-10 donors were collected, pooled, and stored at −80°C.

### Flow cytometric analyses

For flow cytometric analyses, HUVECs were harvested and stained for 20 minutes at 4°C with the following antibodies: anti-PECAM-1-PE-Cy7 (clone WM59, Biolegend, Fell, Germany), anti-ICAM-1-BV421 (clone HA58, BD Pharmingen, Franklin Lakes, USA), anti-VCAM-1-FITC (clone 51-10C9, BD Pharmingen), anti-E-selectin-BV605 (clone 68-5H11, BD Pharmingen), anti- JAM-3-APC (clone SHM33, Biolegend), and anti-VE-cadherin-PE (clone BV9, Biolegend). Viability of HUVECs was analyzed by adding 7-AAD (BD Pharmingen) to the samples 10 minutes prior to analysis. Cells were analyzed using BD LSRFortessa™ or FACSymphony™ (BD Biosciences), BD FACS Diva 8.0.1, and FlowJo software version 10.7.1.

### Analysis of cytokine secretion

For the analysis of cytokine secretion, the cell-free SN from HUVECs were collected. Cytokine concentrations were determined using LEGENDPlex^TM^ Human Inflammation Panel 1 (Biolegend) according to the manufacturer’s instructions and results were analyzed using the LEGENDplex^TM^ data analysis software (Biolegend). The limits of detection are specified for each analyte as follows: hIL-1β: 3.0 pg/ml; hIL-6: 3.3 pg/ml, hIL-8: 3.4 pg/ml; hIL-10: 1.3 pg/ml; hIL-12p70: 1.82 pg/ml; hIL-17A: 1.7 pg/ml; hIL-18: 2.3 pg/ml; hIL-23: 5.1 pg/ml; hIL-33: 3.6 pg/ml; hIFN-α2: 2.3 pg/ml; hIFN-γ: 2.9 pg/ml; hTNF-α: 3.6 pg/ml; hMCP-1: 3.2 pg/ml.

### Vascular leakage assay

For the VL assay, trans-well inserts (0.4 µm pore size, polyethylene terephthalate, translucent, 0.3 cm^2^ growth area) were placed in a 24 well containing 1 ml of ECGM thereby establishing an upper and a lower compartment. To reduce donor-to-donor variations, a pool of HUVECs from four different donors was used. 4x10^4^ HUVECs per trans-well insert were seeded and incubated over night to enable the formation of a confluent monolayer. Subsequently, the indicated stimuli or 8x10^4^ pre-stimulated PBMCs were added to the HUVECs in the upper compartment and incubated for 0, 6, or 24 h until addition of the tracer molecule leading to a total volume of 200 µl in each trans-well to provide comparable hydrostatic pressure. To investigate relative HUVEC-permeability, the flow-through of FITC-dextran (~40 kDA, 6.25 mg/ml, Sigma-Aldrich) after 40 min or FITC-albumin after 90 min (~ 67 kDA, 1.64 mg/ml, MP Biomedicals, Irvine, USA) was determined by quantifying fluorescence intensity (FI) in samples collected from the lower compartment (excitation 491.5−15 nm, emission 528−20 nm; CLARIOstar Plus microplate reader (BMG Labtech, Ortenberg, Germany)). Of note, the initial concentrations of the tracer molecules were determined in previous titration experiments during the establishment phase of the leakage assay to ensure the usage of an adequate amount of tracer molecule within the detection range (data not shown).

An insert without HUVECs served as a control for the maximum tracer molecule flow-through for each individual experiment. Results were analyzed using the MARS software (BMG Labtech). Data were corrected for background by blank (ECGM) subtraction and the relative permeability was calculated by subtracting the FI of unstimulated from stimulated samples in each individual experiment (*FI*_*[stimulated]*_ *– FI*_*[unstim.]*_).

### Correlation studies and statistics

For statistical analyses, the Wilcoxon matched-pairs signed rank test or the Mann-Whitney U test were performed using GraphPad Prism 9.2.0. Correlation analysis between cytokine and flow cytometric markers were performed using the statistic software R v4.2.2. Pairwise correlation between markers were illustrated by scatterplots. Spearman correlation and the respective test for association between markers were performed using pairwise complete observations only.

## Results

### Activation of HUVECs is induced by a broad range of VL-associated stimuli

Until now, studies investigating ECs often mainly focused on a restricted set of parameters for a restricted set of stimuli. At the same time, the responses, with which ECs can contribute to the formation to the development of VL are not fully understood. To approach a comprehensive characterization, we analyzed the effect of 16 different VL-associated stimuli on HUVECs, a well-accepted model of primary ECs used for a broad range of *in vitro* analyses [6,11,15]. First, we investigated HUVEC-activation by analyzing different surface proteins. For this, HUVECs were treated for 6 h, 24 h, and 48 h with cytokines (IL-1β, IL-2, IL-6, IL-8, IFN-α, IFN-γ, or TFN-α), a cytokine cocktail derived from TGN1412-stimulated T cells (SN_TGN1412_) and the respective control (SN_Ø_), vasoactive substances (thrombin, VEGF, bradykinin, or von Willebrand factor (vWF)), and immunological danger signals (LPS, Pam3Cys, or flagellin).

**Fig 1:**
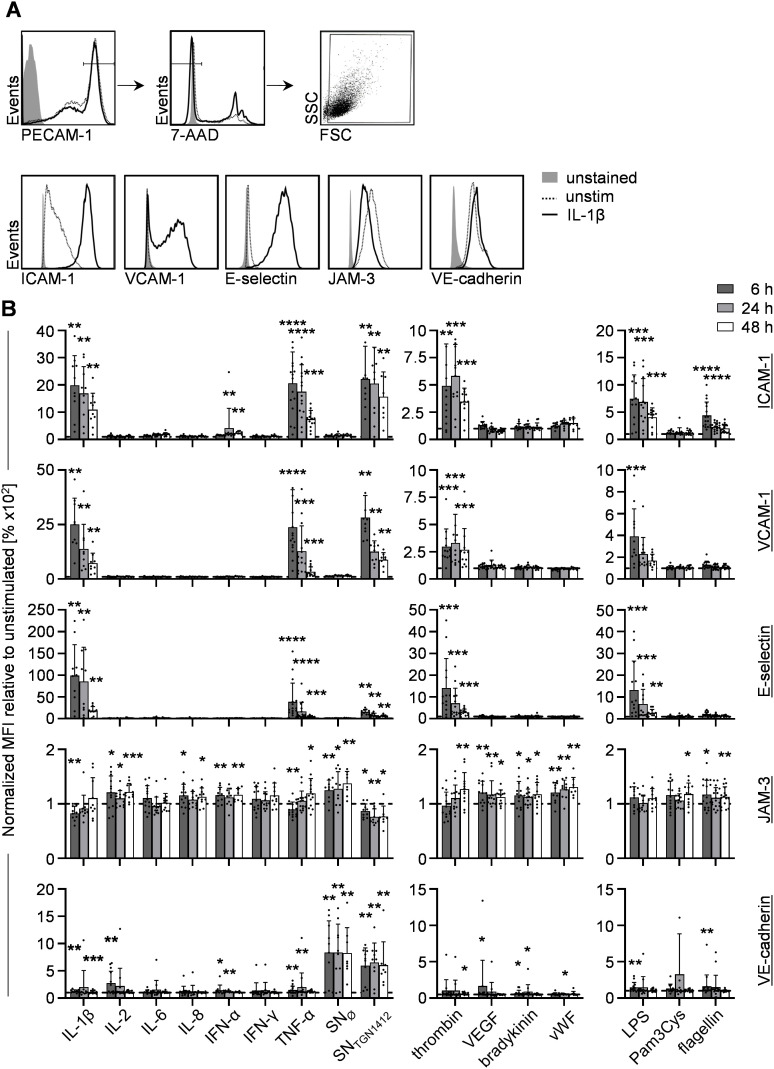
HUVECs are activated upon stimulation with IL-1β, TNF-α, SN_TGN1412_, thrombin, and LPS. 1x10^6^ HUVECs/ml were seeded in 96-well plates, treated with the indicated stimuli or left untreated for 6 h, 24 h, or 48 h, and analyzed by flow cytometry. **(A)** PECAM-1^high^7-AAD^-^ cells were analyzed after excluding cell debris for the expression of ICAM-1, VCAM-1, E-selectin, JAM-3, and VE-cadherin (representative graphs are shown for 1 ng/ml IL-1β (solid line) and untreated HUVECs (unstim., dotted line)). **(B)** Statistical analyses of surface marker-expression normalized to unstimulated HUVEC controls (dotted line) is given for all stimuli and all time points investigated (6 h, dark grey bars; 24 h, light grey bars; 48 h, white bars). Error bars indicate the standard deviation (n = 10-20 from 7 independent experiments). For statistical analyses the Wilcoxon matched-pairs signed rank test was used comparing stimulated samples to to unstimulated controls with *, p ≤ 0.05; **, p ≤ 0.01; ***, p ≤ 0.001; ***, p ≤ 0.0001.

Expression of the apical adhesion molecules ICAM-1, VCAM-1, and E-selectin, known to be upregulated upon HUVEC-activation [17], and the intercellular junctional proteins JAM-3 and VE-cadherin were analyzed on PECAM-1^high^7-AAD^-^ HUVECs by flow cytometry (Fig 1A) using PECAM-1 as a lineage marker for ECs. ICAM-1-, VCAM-1-, and E-selectin-expression was significantly enhanced upon stimulation with IL-1β, TNF-α, SN_TGN1412_, thrombin, and LPS (Fig 1B). Furthermore, IL-1β, TNF-α, and SN_TGN1412_ induced a slight but significant reduction of JAM-3-expression. VE-cadherin-expression on HUVECs was mainly increased upon treatment with SN_TGN1412_ and SN_Ø_ indicating an effect mediated by soluble T cell-specific components independent from TGN1412-stimulation.

**Fig 2:**
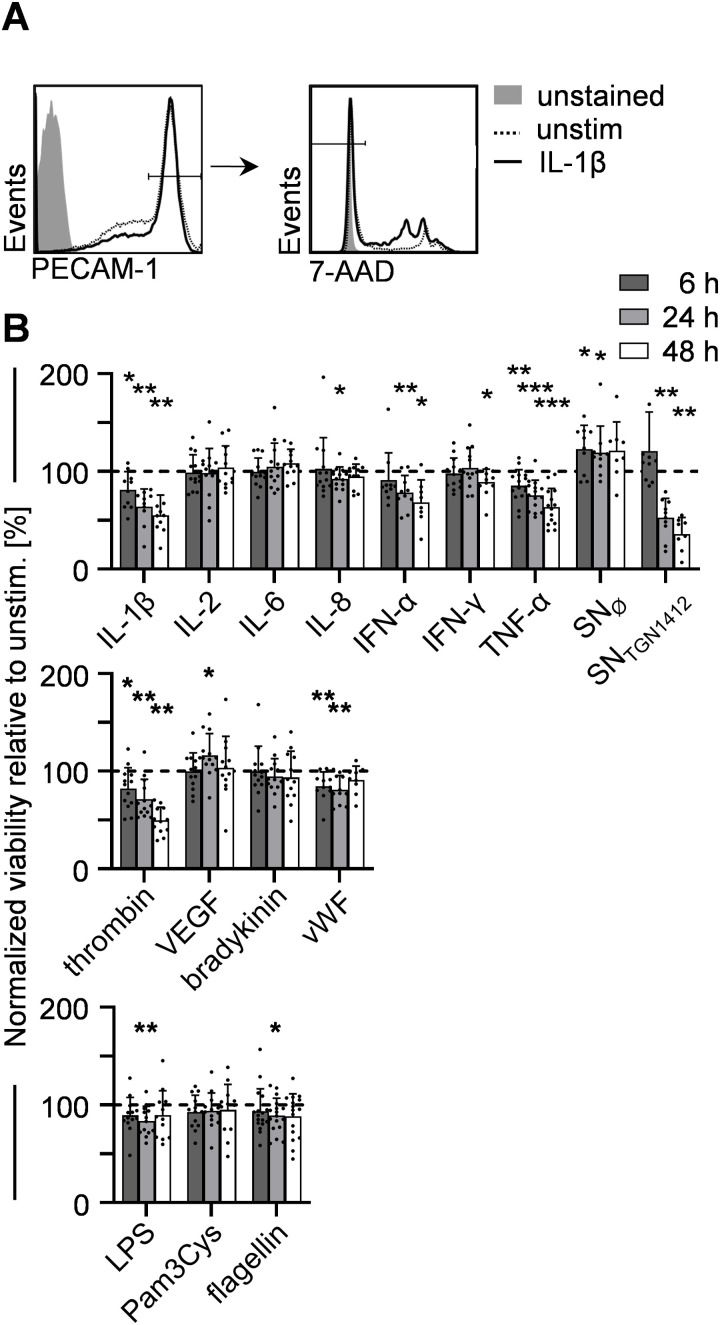
Viability of HUVECs is reduced upon stimulation with IL-1β, IFN-α, TNF-α, SN_TGN1412_, and thrombin. 1x10^6^ HUVECs/ml were seeded in 96-well plates, treated with the indicated stimuli or left untreated for 6 h, 24 h, or 48 h, and analyzed by flow cytometry. **(A)** PECAM-1^high^ cells were analyzed for percentages of 7-AAD^-^ HUVECs (representative graphs are shown for 1 ng/ml IL-1β (solid line) and untreated controls (unstim., dotted line)). **(B)** Statistical analyses of HUVEC-viability normalized to unstimulated HUVECs (dotted line) is given for all stimuli and all time points investigated (6 h, dark grey bars; 24 h, light grey bars; 48 h, white bars). Error bars indicate standard deviations (n = 10-20 from 7 independent experiments). For statistical analyses the Wilcoxon matched-pairs signed rank test was used comparing stimulated samples to unstimulated controls with *, p ≤ 0.05; **, p ≤ 0.01; ***, p ≤ 0.001.

Since (excessive) activation of cells can induce cell-death [18], we analyzed the effect of VL-associated stimuli on HUVEC-viability by quantifying the percentage of 7-AAD^-^ PECAM-1^high^ HUVECs (Fig 2A). Along with the ability to induce strong HUVEC-activation, stimulation with IL-1β, TNF-α, SN_TGN1412_, and thrombin decreased viability of HUVECs over time (Fig 2B). Of note, other compounds such as IFN-α and vWF also significantly reduced HUVEC-viability, despite their inability to induce HUVEC-activation.

**Fig 3:**
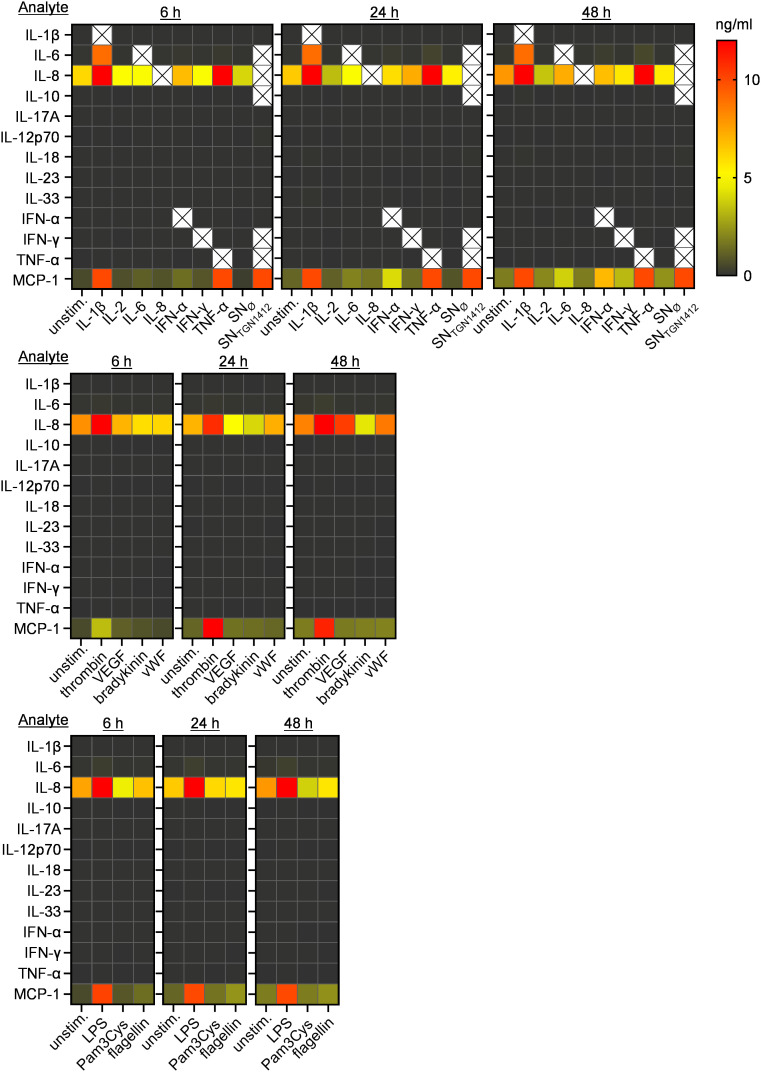
Upon treatment with IL-1β, IFN-α, TNF-α, SN_TGN1412_, or thrombin, HUVECs secrete IL-8, MCP-1, and IL-6. 1x10^6^ HUVECs/ml were seeded in 96-well plates, treated with the indicated stimuli or left untreated for 6 h, 24 h, or 48 h. Secretion of cytokines was analyzed using a human inflammation panel bead-based multiplex assay (Biolegend). The level of each cytokine analyzed is given in heatmaps ranging from 0 ng/ml (black) to 12 ng/ml (red). Mean values in numbers and statistical analyses using Wilcoxon matched-pairs signed rank test are additionally provided in Table 2 (n = 3-8 from 8 independent experiments). Cytokines which were used for the respective stimulation were not analyzed and are marked with a black cross on white ground.

ECs are capable of secreting cytokines and chemokines and their role in the development of a cytokine storm has been discussed repeatedly [6,12,13]. Using a multiplex assay, we analyzed whether the different VL-associated compounds induce cytokine production by HUVECs (Fig 3). Interestingly, HUVECs responded with a restricted, stimulus-unspecific set of two chemokines and one cytokine. IL-8, a chemokine known to be secreted by ECs, is already detectable in the SN of untreated HUVECs [19,20]. Treatment with IL-1β, TNF-α, SN_TGN1412_, thrombin, or LPS induced elevated IL-8- and monocyte chemoattractant protein (MCP)-1-levels. For all other stimuli, no or only minor increased secretion of IL-8 and/or MCP-1 was detected. Several stimulations, e.g., IL-1β and to a much lower extent LPS (see Table 2) also induced IL-6 secretion. All other cytokines analyzed were detected in very low amounts or below the detection limit of the assay, as shown by the raw data of the cytokine analyses depicted in Table 2.

**Table 2:** Mean values and standard deviations of measured cytokines and chemokones secreted by HUVECs upon treatment with indicated stimuli.

6 h (pg/ml)																											
Analyte	IL-1β		IL-6		IL-8		IL-10		IL-12-p70		IL-17A		IL-18		IL-23		IL-33			IFN-α		IFN-γ		TNF-α		MCP-1 (CCL2)	
**Stimulus**	Mean	SD	Mean	SD	Mean	SD	Mean	SD	Mean	SD	Mean	SD	Mean	SD	Mean	SD	Mean		SD	Mean	SD	Mean	SD	Mean	SD	Mean	SD
unstim	1.2	0.7	75.3	51.5	6712.9	2902	0.4	0.4	1.0	0.4	0.0	0.1	3.2	1.0	1.3	0.4	7.6		6.2	0.4	0.3	0.3	0.3	0,3	0.5	676.2	417.0
IL-1β	X	X	9000	0.0	12000	0.0	17.6	5.2	8.4	2.1	0.6	0.2	22.3	3.1	7.4	1.0	24.7		6.4	4.9	1.0	3.2	0.8	4,8	2.2	10000	0.0
IL-2	0.9	0.8	63.4	40.4	4423.2	1437.7	0.1	0.3	0.7	0.5	0.0	0.1	2.4	1.0	0.8	0.6	6.3		6.7	0.4	0.3	0.2	0.3	0,5	0.5	604.6	305.1
IL-6	27.6	62.8	X	X	5785.1	3209.5	16.9	2.0	5.4	1.0	0.4	0.1	8.4	2.6	4.7	0.7	14.0		6.1	3.0	0.6	2.9	1.3	1,4	1.4	1672.0	1673.4
IL-8	2.8	1.3	54.4	19.0	X	X	0.4	0.5	1.8	0.8	0.2	0.1	5.3	2.5	2.0	0.9	12.6		13.7	0.6	0.4	0.7	0.5	1,0	0.9	730.5	469.0
IFN-α	1.7	0.6	155.4	83.0	6853.5	3194.5	0.7	0.7	4.1	0.9	0.1	0.1	5.6	1.1	2.2	0.3	6.4		0.8	X	X	1.1	0.8	0.4	0.5	1824.1	1097.0
IFN-γ	6.0	11.1	841.7	1736.8	4811.2	491.6	1.1	2.1	1.1	0.7	0.0	0.1	3.2	1.2	1.2	0.6	7.7		5.9	0.6	0.5	78.5	84.4	0.6	0.6	795.7	151.3
TNF-α	7.2	5.2	166.1	91.6	9489.3	3258.6	0.4	0.4	2.1	0.7	0.2	0.1	11.9	7.4	2.6	1.0	10.8		3.7	1.8	1.0	1.0	0.3	X	X	9393.9	1487.8
SN_Ø_	0.5	0.4	25.8	6.7	3905.3	1071.5	0.0	0.0	0.4	0.3	0.0	0.0	1.8	0.3	0.5	0.5	4.3		4.0	4.8	8.2	0.8	0.3	0.0	0,0	392.5	220.8
SN_TGN1412_	7.3	1.0	X	X	X	X	X	X	6.2	1.0	25.8	1.8	12.7	3.1	5.7	0.8	10.4		2.8	2.8	0.3	X	X	X	X	100000	0.0
thrombin	2.0	0.4	142.4	58.0	6817.9	2462.2	0.5	0.5	1.1	0.3	0.1	0.1	4.2	0.7	1.3	0.3	8.4		5.5	0.7	0.4	0.6	0.4	0.7	0,7	3097.6	1060.5
VEGF	1.1	0.7	73.9	52.2	5312.1	1740.8	0.3	0.5	0.9	0.2	0.0	0.0	2.9	0.6	0.6	0.6	8.1		5.4	0.4	0.3	0.6	0.3	0.6	0,6	822.6	480.1
bradykinin	0.7	0.8	58.6	44.4	4636.6	1822.0	0.0	0.0	0.8	0.2	0.0	0.0	4.5	4.3	0.8	0.5	6.1		6.6	0.4	0.3	0.1	0.3	0.6	0,5	645.2	437.8
vWF	0.9	0.3	52.4	28.2	6496.0	3152.9	0.1	0.2	0.5	0.4	0.0	0.0	2.5	0.7	0.8	0.5	2.4		2.2	0.4	0.6	0.2	0.3	0.1	0,3	693.9	378.8
LPS	3.2	0.5	239.1	117.8	8707.3	1875.6	0.7	0.4	1.5	0.2	0.2	0.0	6.4	0.8	1.9	0.3	9.6		7.0	1.0	0.6	0.7	0.0	1.0	0,6	8057.4	1803.1
Pam3Cys	0.7	0.9	51.3	34.4	4121.8	1810.7	0.1	0.3	0.8	0.3	0.0	0.0	2.4	1.1	1.0	0.3	6.6		7.7	0.4	0.2	0.3	0.4	0.2	0,5	665.2	464.9
flagellin	1.0	0.8	64.8	38.7	5277.7	1767.6	0.1	0.3	0.8	0.2	0.0	0.1	3.2	1.1	0.9	0.5	6.0		5.9	0.3	0.3	0.3	0.3	0.2	0,4	1322.3	763.0
**24 h (pg/ml)**																											
**Analyte**	IL-1β		IL-6		IL-8		IL-10		IL-12-p70		IL-17A		IL-18		IL-23		IL-33			IFN-α		IFN-γ		TNF-α		MCP-1 (CCL2)	
**Stimulus**	Mean	SD	Mean	SD	Mean	SD	Mean	SD	Mean	SD	Mean	SD	Mean	SD	Mean	SD	Mean		SD	Mean	SD	Mean	SD	Mean	SD	Mean	SD
unstim	1.1	0.7	76.4	37.2	6699.8	2453.0	0.4	0.3	0.8	0.4	0.0	0.1	3.4	1.0	0.5	0.7	5.5		4.1	0.5	0.4	0.3	0.4	0.6	0.5	1144.0	530.3
IL-1β	X	X	9000	0.0	12000.0	0.0	20.8	4.5	11.1	2.4	0.8	0.2	28.9	5.7	10.2	2.4	26.4		3.3	6.3	1.4	4.4	1.0	4.3	0.7	10000	0.0
IL-2	0.8	0.7	51.7	55.0	4144.6	1856.1	0.1	0.3	0.7	0.1	0.0	0.0	2.4	0.3	0.2	0.5	3.8		5.2	0.4	0.3	0.1	0.2	0.3	0.5	996.2	577.6
IL-6	2.7	0.8	X	X	5236.0	2154.2	18.3	2.3	5.8	0.8	0.4	0.0	9.5	1.9	5.0	1.0	14.6		4.8	3.4	0.4	2.4	0.7	2.0	0.3	2689.4	1625.1
IL-8	3.1	1.0	66.1	44.9	X	X	0.7	0.2	2.0	0.7	0.2	0.1	6.2	1.8	1.9	1.3	11.4		5.3	1.1	1.0	1.2	0.7	1.3	0.7	1675.9	1130.6
IFN-α	1.9	0.6	176.4	84.8	7102.8	2761.5	1.1	0.1	3.7	0.4	0.1	0.1	6.0	1.1	0.8	1.1	6.9		1.8	X	X	0.8	0.6	0.9	0.2	3976.4	2580.3
IFN-γ	1.4	0.8	84.2	69.8	6337.6	1698.6	0.4	0.4	0.9	0.3	0.0	0.1	3.7	1.1	0.6	0.8	5.2		7.2	1.2	1.6	87.1	94.6	0.4	0.6	1544.0	899.9
TNF-α	8.1	2.6	307.3	201.0	11556.9	756.8	1.1	0.3	3.4	1.1	0.3	0.1	15.1	3.6	4.1	1.5	11.3		3.7	2.5	0.3	2.9	3.6	50.7	51.6	8936.5	3008.0
SN_Ø_	0.7	0.6	58.5	36.4	5844.9	2970.1	0.0	0.0	0.4	0.4	0.0	0.0	2.4	0.7	0.2	0.6	0.0		0.0	0.3	0.4	0.1	0.3	0.2	0.2	2869.5	4038.3
SN_TGN1412_	14.2	3.5	X	X	X	X	X	X	15.3	2.9	11.3	6.0	31.3	5.4	14.0	3.3	21.5		5.2	7.3	0.8	X	X	X	X	10000	0.0
thrombin	4.9	2.0	215.0	167.2	9762.3	2115.8	0.7	0.5	2.0	1.1	0.2	0.1	9.9	3.7	2.1	1.7	10.5		8.3	1.5	1.0	1.0	0.4	1.5	1.0	10000	0.0
VEGF	1.0	1.0	81.5	63.9	5721.3	2412.1	0.4	0.4	0.8	0.5	0.0	0.1	3.2	0.8	0.8	0.7	6.4		6.4	0.5	0.3	0.4	0.3	0.4	0.5	1161.1	561.0
bradykinin	0.8	1.1	64.0	38.7	5858.4	3362.4	0.3	0.4	0.8	0.6	0.0	0.1	3.3	1.1	0.8	0.7	6.9		5.5	0.5	0.3	0.5	0.5	0.4	0.6	1378.3	765.4
vWF	0.9	0.6	74.3	47.8	7071.4	2048.8	0.1	0.2	0.6	0.4	0.0	0.0	3.1	0.6	0.2	0.5	3.1		3.4	0.3	0.4	1.0	1.6	0.4	0.4	1136.5	672.6
LPS	6.2	1.0	300.8	156.3	11769.0	425.3	1.1	0.4	2.4	0.7	0.2	0.1	11.2	1.7	2.3	1.5	12.0		4.7	2.0	0.3	1.1	0.2	1.5	0.9	10000	0.0
Pam3Cys	1.1	0.6	66.6	53.5	5609.0	1438.7	0.3	0,4	0.8	0.2	0.0	0.0	3.2	0.5	0.5	0.7	6.0		6.1	0.6	0.4	0.4	0.4	0.2	0.4	1481.9	752.5
flagellin	1.5	1.7	84.2	61.9	6519.9	2679.8	0.3	0.4	1.0	0.7	0.0	0.1	4.2	2.3	0.7	1.1	5.7		6.6	0.5	0.6	0.3	0.5	0.7	0.7	2841.9	2585.5
**48 h (pg/ml)**																											
**Analyte**	IL-1β		IL-6		IL-8		IL-10		IL-12-p70		IL-17A		IL-18		IL-23		IL-33			IFN-α		IFN-γ		TNF-α		MCP-1 (CCL2)	
**Stimulus**	Mean	SD	Mean	SD	Mean	SD	Mean	SD	Mean	SD	Mean	SD	Mean	SD	Mean	SD	Mean		SD	Mean	SD	Mean	SD	Mean	SD	Mean	SD
unstim	3.3	5.2	156.8	178.5	7476.6	2744.6	0.2	0.2	1.0	0.7	0.0	0.1	4.3	2.8	0.9	1.1	3.5		4.3	0.5	0.7	1.1	1.6	0.2	0.3	3036.0	3169.7
IL-1β	X	X	9000	0.0	12000	0.0	29.0	1.1	12.4	0.6	0.9	0.0	30.5	1.0	12.1	1.0	20.3		0.8	6.5	0.6	10.5	9.0	4.7	0.2	10000.0	0.0
IL-2	2.1	1.8	71.7	26.5	4788.4	2007.3	0.0	0.0	0.7	0.5	0.0	0.1	3.3	1.4	0.7	0.7	4.3		4.8	0.1	0.3	0.4	0.4	0.0	0.0	2037.3	1136.4
IL-6	40.7	91.1	X	X	7581.7	3096.2	21.0	11.1	7.6	3.7	0.5	0.3	14.4	9.6	6.6	4.5	13.0		8.1	2170.4	5304.5	3.5	2.7	2.4	1.7	5447.6	3632.8
IL-8	3.8	1.9	91.0	41.0	X	X	0.1	0.2	2.0	1.1	0.2	0.1	6.8	3.1	2.0	1.8	7.0		4.9	3.7	8.0	0.9	0.6	0.6	0.9	2145.3	1029.7
IFN-α	2.2	0.4	190.8	77.3	7841.9	2461.8	0.8	0.2	2.4	1.1	0.0	0.1	5.3	1.2	0.5	0.7	4.8		3.0	X	X	0.8	0.6	0.3	0.5	4934.0	3207.5
IFN-γ	24.2	51.1	1878.7	3981.2	6664.2	3372.8	3.0	6.4	2.0	3.0	0.1	0.3	6.9	7.3	1.9	3.1	5.2		6.6	0.9	1.7	X	X	0.9	1.7	3892.0	3564.6
TNF-α	15.5	15.4	601.0	451.5	11683.3	838.0	1.6	1.0	4.2	1.3	0.4	0.1	18.2	4.1	5.4	1.8	11.6		3.4	2.8	0.6	2.0	0.7	X	X	10000	0.0
SN_Ø_	0.8	0.9	58.1	14.7	6709.0	3777.3	0.1	0.2	0.3	0.4	0.0	0.0	2.6	1.1	0.0	0.0	0.0		0.0	0.3	0.4	0.1	0.2	0.3	0.3	2193.1	1105.5
SN_TGN1412_	13.5	4.1	X	X	X	X	X	X	13.4	3.2	5.5	4.2	28.9	6.6	13.0	3.5	18.8		1.7	7.5	1.9	X	X	X	X	10000	0.0
thrombin	5.6	2.1	269.2	104.1	10213.6	2108.8	1.3	1.5	2.0	1.2	0.2	0.1	9.9	3.8	2.2	1.8	7.7		6.1	1.8	0.4	10.1	18.7	0.7	1.0	9471.8	1181.1
VEGF	11.6	21.4	458.0	747.3	8313.7	3832.3	0.9	1.3	1.3	1.2	0.1	0.1	5.0	4.5	1.3	1.7	5.4		3.4	4.3	8.3	1.2	1.2	0.5	0.9	3099.8	3930.9
bradykinin	1.0	0.6	65.6	35.7	4526.8	1724.7	0.1	0.2	0.5	0.3	0.0	0.0	2.6	0.9	0.6	0.5	4.4		3.1	0.2	0.4	0.2	0.3	0.2	0.3	1685.9	560.6
vWF	1.2	0.8	87.4	12.0	7809.4	2957.5	0.3	0.2	0.6	0.3	0.0	0.0	3.4	0.9	0.3	0.6	0.8		1.8	0.3	0.5	0.1	0.3	0.1	0.2	1843.2	1008.3
LPS	6.0	1.8	394.0	187.8	12000	0.0	1.1	1.0	2.4	1.1	0.2	0.2	11.9	4.5	2.2	2.0	8.5		2.4	2.8	1.9	12.3	21.9	1.6	0.5	10000	0.0
Pam3Cys	1.2	0.8	67.2	31.0	4455.1	1938.2	0.0	0.0	0.6	0.4	0.0	0.0	3.0	1.1	0.6	0.6	3.9		2.7	0.6	0.9	0.3	0.3	0.2	0.4	1924.3	786.6
flagellin	1.6	1.3	83.1	43.8	5946.4	1842.3	0.1	0.2	0.7	0.5	0.1	0.1	3.6	1.8	0.6	0.8	3.3	3.5		3.7	9.0	0.4	0.5	0.2	0.5	2955.0	2741.1

X = Present in input

Collectively, HUVEC-activation can be observed mainly upon stimulation with IL-1β, TNF-α, SN_TGN1412_, thrombin, and LPS, characterized by upregulation of ICAM-1, VCAM-1, and E-selectin, reduced HUVEC-viability, and, less pronounced, downregulation of JAM-3. In addition, treatment of HUVECs with IL-1β, TNF-α, SN_TGN1412_, thrombin, and LPS resulted in enhanced levels of mainly IL-8 and MCP-1.

In order to analyze the relationship of these results, we performed pairedSpearman correlation studies (surface marker with surface marker, Fig A in S1 Fig; cytokine with cytokine, Fig B in S1 Fig; and surface marker with cytokine, Fig C in S1 Fig). The expression level of ICAM-1, VCAM-1, and E-selectin as well as the secretion of IL-6, IL-8, and MCP-1 show moderate to strong positive correlations in all possible combinations. In turn, expression of JAM-3 and VE-cadherin weakly correlate, whereas HUVEC-viability does not correlate with apical marker expression nor chemokine/cytokine secretion. Collectively, the positive correlations between activation markers and cytokine expression draw a picture of a fully activated HUVEC culture upon adequate stimulation.

### Treatment of HUVECs with VL-associated stimuli does not necessarily result in increased relative permeability *in vitro*

**Fig 4:**
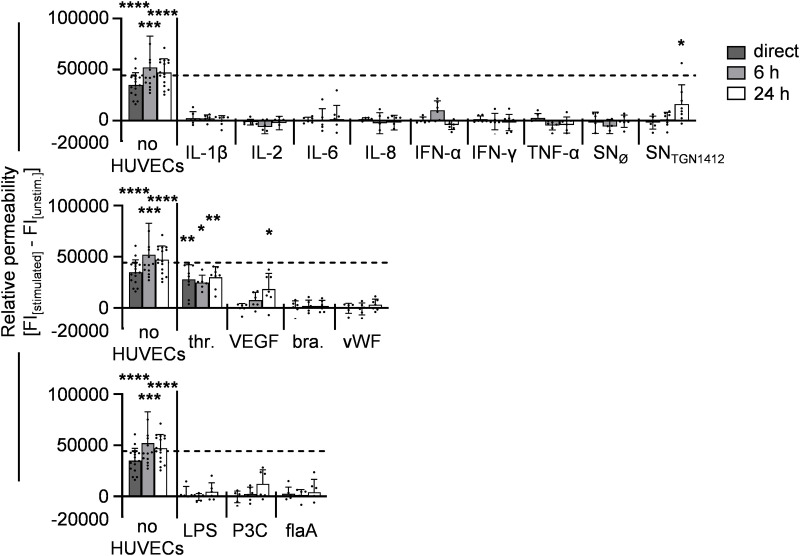
Direct treatment of HUVECs with VL-associated stimuli does not necessarily result in increased relative permeability *in vitro.* 4x10^4^ HUVECs were seeded into trans-well inserts placed in medium-filled 24-well-plates, incubated overnight, and then treated with indicated stimuli or left untreated. The flow-through of FITC-albumin across HUVEC-monolayers within 90 minutes into the lower compartment of the trans-well system was analyzed directly upon co-incubation (dark grey bars), upon 6 h (light grey bars), or upon 24 h (white bars) incubation (timepoints were measured within individual experiments). The fluorescence was determined at 528-520 nm in 100 µl-samples from the lower chamber. A cell-free trans-well, representing the maximum possible flow-through (dotted line), served as control. Relative permeability was calculated by subtraction of FI_[unstim.]_ from FI_[stimulated]_;(thr. = thrombin; bra. = bradykinin; flaA = flagellin). Error bars indicate standard deviations (n = 6-19 from 14-19 independent experiments). For statistical analyses the Wilcoxon matched-pairs signed rank test was used comparing stimulated samples to unstimulated controls with *, p ≤ 0.05; **, p ≤ 0.01; ****, p ≤ 0.0001.

In order to investigate the effect of VL-associated compounds on HUVEC-integrity, trans-well-based VL-assays were performed. We analyzed the flow-through of FITC-Albumin (~ 67 kDa) across HUVEC-monolayers upon direct stimulation or after 6 h or 24 h of incubation with the indicated treatments (Fig 4). Values from untreated controls were subtracted from stimulated samples for each individual experiment. As expected, thrombin significantly elevated relative permeability [9]. A massive pro-inflammatory milieu can result in the formation of VL in patients [2,12,21]. Accordingly, treatment of HUVECs with SN_TGN1412_ increased relative permeability upon 24 h of stimulation. Since VEGF is known to detach inter-endothelial VE-cadherin interaction [3,10,22], the significant VEGF-mediated increase in relative HUVEC-permeability we observed 24 h post incubation was expected. Interestingly, stimulation with single cytokines, further vasoactive substances, or immunological danger signals did not affect relative HUVEC-permeability, irrespective of their impact on HUVEC-activation or -viability.

In conclusion, only a limited number of stimuli, namely thrombin, VEGF, and SN_TGN1412_, directly increased relative HUVEC-permeability. While IL-1β-, TNF-α-, and LPS-stimulation induced HUVEC-activation and reduced viability, this did not result in elevated relative permeability. Hence, HUVEC-activation is not inevitably associated with enhanced relative permeability and *vice versa*.

### Immune cells are required for IL-1β- and LPS-mediated relative HUVEC-permeability

**Fig 5:**
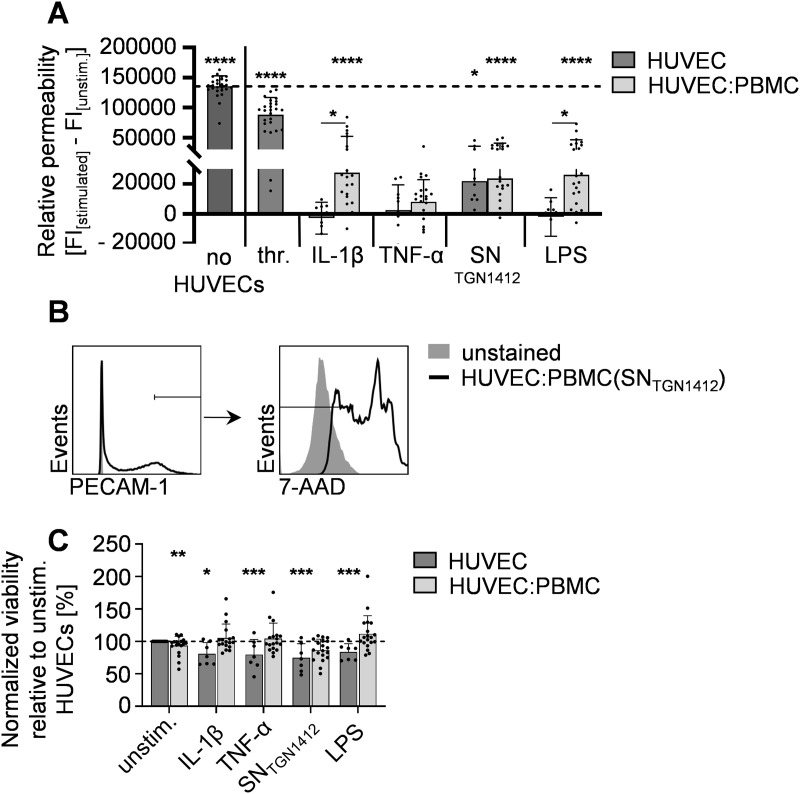
Immune cells are required for IL-1β- and LPS-mediated increase in relative HUVEC-permeability. Human PBMCs were isolated from buffy coats, seeded in 24-well plates at a concentration of 1x10^6^ cells/ml, and pre-stimulated with IL-1β, TNF-α, SN_TGN1412_, LPS, or left untreated for 5 d. 4x10^4^ HUVECs were seeded in trans-well inserts placed in medium-filled 24-well-plates at and incubated overnight, and then co-cultured with 8x10^4^ pre-stimulated PBMCs or treated with the indicated stimuli for 24 h as control. Unstimulated HUVECs alone or unstimulated HUVEC:PBMC co-cultures served as controls. **A)** The flow-through of FITC-dextran-flux across HUVEC-monolayers within 40 minutes into the lower compartment of the trans-well system was analyzed for HUVEC-monocultures (dark grey bars) or HUVEC:PBMC co-cultures (light grey bars). The fluorescence was determined in 100 µl-samples from the lower chamber at 528−520 nm. An empty trans-well, representing the maximum possible flow-through (dotted line), served as control . Relative permeability was calculated by subtraction of FI_[unstim.]_ from FI_[stimulated]_. Error bars indicate standard deviations (n = 10−26 from 4−14 independent experiments). For statistical analyses the Mann-Whitney U test was used comparing stimulated samples to unstimulated controls with *, p ≤ 0.05; ****, p ≤ 0.0001. **(B)** PECAM-1^high^ cells were analyzed for percentages of 7-AAD^-^ HUVECs within HUVEC:PBMC-co-cultures (representative graph shown for SN_TGN1412_-treated HUVEC:PBMC-co-culture). **(C)** Statistical analyses of HUVEC-viability normalized to unstimulated HUVECs (dotted line) is given for all co-cultures investigated. Error bars indicate the standard deviation (n = 19 from 13 independent experiments). For statistical analyses the Wilcoxon matched-pairs signed rank test was used (*, p ≥ 0.05).

Peripheral blood cells continuously interact with the vascular endothelium. Additionally, immune cells are described to be involved in the development of VL [6,7,11]. To analyze, whether immune cells promote relative HUVEC-permeability, we pre-stimulated PBMCs for 5 days with the immunological stimuli inducing HUVEC-activation (see Figs 1 and 3): IL-1β, TNF-α, LPS, or SN_TGN1412_. Since VEGF and thrombin are no classical immunological stimuli and induce increased permeability alone (Fig 4), these treatments were not used for PBMC-pre-stimulation. Pre-stimulated PBMCs were co-cultured with HUVECs within the VL-assay described above for 24 h. Here, thrombin-treated HUVECs served as positive-control for increased relative permeability. IL-1β- and LPS-pre-stimulated PBMCs significantly increased relative permeability of HUVECs compared to untreated HUVEC-mono- and co-cultures as well as to HUVEC-monocultures treated with IL-1β or LPS. In contrast, TNF-α-pre-stimulated PBMCs did not significantly increase relative permeability. The co-cultivation with SN_TGN1412_-treated PBMCs increased relative HUVEC-permeability when compared to untreated co-cultures. Nevertheless, increased permeability upon treatment with SN_TGN1412_ alone was not significantly elevated by SN_TGN1412_-treated PBMCs, suggesting a direct effect of SN_TGN1412_ on relative HUVEC-permeability, which is not exacerbated by the addition of immune cells *in vitro*. To unravel if the co-culture with PBMCs affects HUVEC-viability, 7-AAD^-^PECAM-1^high^ HUVECs within the co-cultures were determined (Fig 5B) and compared to accordingly treated HUVEC-monocultures after normalization to untreated HUVECs (Fig 5C). Of note, untreated PBMCs slightly decreased HUVEC-viability. Interestingly, while the single stimuli decreased HUVEC-viability similarly as observed in Fig 2, HUVEC-viability was not affected by IL-1β-, TNF-α-, SN_TGN1412_-, and LPS-stimulated PBMCs as compared to untreated HUVEC-monocultures (Fig 5C). Thus, the PBMC-mediated increase in relative HUVEC-permeability is not associated with reduced HUVEC-viability.

Taken together, these data demonstrate that immune cells play a critical role for the formation of VL, which is not necessarily associated with decreased HUVEC-viability. Moreover, not all immunological stimuli enabled the PBMCs to enhance relative permeability suggesting that there are different mechanisms by which immune-mediated VL is driven.

## Discussion

Both EC-biology and VL have been a subject of research since decades [1,3,17,23,24]. Yet, the mechanisms behind the development of pathologically increased permeability, including the tipping point between physiological permeability and pathological leakage, are not fully understood.

Endothelial activation markers as well as several EC-associated cytokines have been analyzed in various studies, but often with focus on one stimulus and only with a restricted set of readouts. This broad characterization study takes different types of VL-associated compounds and several readouts into account for each donor analyzed to generate a coherent set of data. In this study, we observed that the HUVECs activated by different stimuli respond with a largely conserved set of activation markers: increased ICAM-1-, VCAM-1-, and E-selectin-expression, elevated levels of IL-8, MCP-1, and, for potent stimuli, additionally IL-6. The consideration of further activation markers for ECs, such as Ca^2+^-mobilization, NO production, thrombomodulin expression, or additional surface molecules like P-Selectin could provide further clarification of potential stimulus-specific responses of HUVECs.

Thrombin-induced increased expression of adhesion molecules and increased relative HUVEC-permeability immediately upon stimulation. In contrast, treatment of HUVECs with single cytokines such as IL-1β or TNF-α resulted in an increase of ICAM-1-, VCAM-1-, and E-selectin as well, but did not elevate relative permeability (Fig 4) showing that activation of HUVECs does not necessarily result in increased relative permeability. However, incubation of HUVECs with SN_TGN1412_ increased relative permeability indicating that an accumulation of cytokines enables the formation of VL. Accordingly, a cytokine storm induced by different etiologies, such as sepsis, infections, or during immunotherapies, are related with the development of VL [2,8,12,13,23]. Furthermore, sera of patients with Clarksons’s Disease are characterized by elevated serum levels of IL-1β, IL-6, IL-8, IL-12, TNFα, C-X-C motif ligand (CXCL)10, and CC-chemokine ligand (CCL)2 (MCP-1) [25].

Different cytokines as part of pro-inflammatory immune responses are all potential key players during the formation of VL. *In vivo* studies demonstrated that the IL-1β receptor antagonist Anakinra® can ameliorate inflammation- and leakage-mediated acute respiratory distress syndrome induced by SARS-CoV2-infection [8]. Interestingly, SN_TGN1412_ can elevate relative HUVEC-permeability, despite the absence of IL-1β in this cytokine cocktail [15]. Thus, apparently different cytokine compositions are able to culminate in the formation of VL. With regards to the few cytokines measured upon HUVEC-activation (Fig 3), immune cells are likely to play the dominant role during the formation of cytokine storms and cytokine-driven VL. Activated ECs can upregulate expression of cytokine receptors. Additionally, instead of directly inducing EC-activation, several stimuli increase the responsiveness of ECs towards certain cytokines. As supported by our findings (Fig 1), IFN-γ was shown to induce only minor EC-activation. However, it can enhance TNF-α-mediated effects on ECs including increased permeability [11,26,27]. Within SN_TGN1412_, several cytokines (e.g., IFN-γ, TNF-α, IL-6, IL-8, or IL-10) [15] potentially contribute to the development of VL. Thus, the observed elevated relative HUVEC-permeability was probably driven by more than IFN-γ-mediated increased responsiveness towards TNF-α. The identification of key cytokines or maybe even “shared” cytokine-combinations causing VL remains an open challenge.

ECs are termed “acquired immune cells”, since they can express molecules, which are involved in innate and adaptive immune responses [6]. They express pattern recognition receptors such as CD14 and toll-like-receptor (TLR)-4 recognizing LPS [6], which goes along with the LPS-mediated effects on HUVECs observed in this study. Furthermore, stimulated ECs can upregulate co-stimulatory molecules and thus facilitate interaction with T cells. It was shown that TGN1412-induced T cell proliferation can be mediated by T cell:HUVEC interaction via inducible T-cell co-stimulator ligand (L-ICOS)-engagement with ICOS [15]. Thus, endothelial activation might impact immune responses and therefore potentially promote the development of VL.

Pro-inflammatory conditions are able to immediately intensify the continuous contact of immune cells with the endothelium driven by several surface molecules [2,5,6,28,29]. In line with the literature, activated HUVECs, as analyzed in this study, upregulate ICAM-1, VCAM-1, and E-Selectin, known for EC:immune cell-interaction, and secrete chemokines, known to recruit immune cells (Figs 1, 3, and Figs A, B, and C in S1 Fig) [2,3,5,6,10]. Interestingly, interaction of ICAM-1 expressed by HUVECs with lymphocyte function-associated antigen-1 on immune cells was found to be a critical factor for increased permeability *in vitro* [28].

For our study, we used HUVECs as EC-model but are aware of the diversity of EC-types, which are specialized for organ-specific requirements. Interestingly, in different organs mainly ICAM-1 but also VCAM-1 are described as critical molecules facilitating immune cell-interaction and -transmigration into the underlying tissue [30,31]. Thus, despite the heterogenicity of ECs, the insights obtained here might be applicable to other EC-types and shared cellular interaction-mechanisms could serve as treatment-targets for VL.

The demand for models and their capability to reflect pathologies with sophisticated immunological background, including VL and CRS as “upstream-pathology” are broadly discussed [1,12,13,32]. Despite the limitations of 2D leakage assays, as also applied by others, such as the lack of blood-flow or the removal of dead cells, valuable insights can be obtained with the assay used in this study. The connection of the permeability assay with further readouts, such as HUVEC activation markers or viability, especially when including immune cells, will contribute to further unravel mechanisms behind VL, which could be verified with supporting approaches, e.g., vessel-on-a-chip models [33].

It has been reported that immune cells promote EC-permeability. For example, monocytes can enhance adhesion and transmigration of T cells on and across HUVEC-monolayers [34]. Furthermore, during, e.g., malaria disease, VL is associated with EC-damage mediated by antigen-specific cytotoxic T cells [35]. Due to the absence of a foreign antigen during pre-stimulation of the PBMCs in our study-set-up, rather activated bystander T cells than antigen-specific cytotoxic T cells might contribute to the observed increased permeability. In our *in vitro* assay, PBMCs pre-treated with IL-1β and LPS increased relative permeability of unstimulated HUVEC-monolayers, while TNF-α-pre-treated PBMCs did not (Fig 5). Thus, for certain immunological stimuli, immune cells play a critical role in the development of VL. Nevertheless, potential different, stimulus-specific mechanisms and also effects of immune cells on activated ECsremain to be elucidated.

With our data we draw a coherent picture of activated HUVECs, with a conserved, stimulus-independent activation-marker-pattern. This EC-activation is not necessarily associated with increased relative HUVEC-permeability. Furthermore, depending on the stimulus, elevated relative permeability is enhanced by immune cells. With the established *in vitro* assay, we can provide comprehensive analyses to unravel (immune-mediated) mechanisms which contribute to the formation of VL. For future studies, the dissection of cell-mediated and cytokine-mediated effects will deliver important mechanistic understanding behind this severe pathology as important step towards better options for treatment, diagnosis, or even prevention of VL.

## Supporting information

S1 Fig Expression of ICAM-1, VCAM-1, E-Selectin, IL6, IL-8, and MCP-1 show a positive correlation. Data for ICAM-1, VCAM-1, and E-Selectin (Fig 1) and IL-6, IL-8, and MCP-1 (Fig 3) were analyzed using Spearman correlation pairing all combinations with the same underlying conditions. (A) Spearman correlation results pairing ICAM-1, VCAM-1, and E-selectin. (B) Spearman correlation results pairing of IL-6, IL-8, and MCP-1. (C) Spearman correlation results pairing ICAM-1, VCAM-1, and E-selectin with IL-6, IL-8, and MCP-1. The correlation coefficient (r) was defined as: r < 0.5 low positive correlation; r < 0.7 moderate positive correlation; r < 0.9 high positive correlation.
(PDF)

S1 File 250704_Data _Gogesch and Ortega Iannazzo et al_revised. (XLSX)

## Abbreviations

Ca2+: calcium
CRS: cytokine release syndrome
EC: endothelial cell
FI: fluorescence intensity
HUVECs: human umbilical vein endothelial cells
ICAM: intercellular adhesion molecule
IFN: interferon
IL: interleukin
JAMs: junctional adhesion proteins
LPS: lipopolysaccharide
MCP: monocyte chemoattractant protein
NO: nitric oxide
PBMCs: peripheral blood mononuclear cells
SN: supernatant
TLR: toll-like-receptor
TNF: tumor necrosis factor
VCAM: vascular cell adhesion molecule
VE: vascular endothelial
VEGF: vascular endothelial growth factor
VL: vascular leakage
vWF: von Willebrand factor

## References

[1] Bichon A, Bourenne J, Gainnier M, Carvelli J. Capillary leak syndrome: state of the art in 2021. Rev Med Interne. 2021;42(11):789–96. doi: 10.1016/j.revmed.2021.05.012 34099313

[2] Duan C-Y, Zhang J, Wu H-L, Li T, Liu L-M. Regulatory mechanisms, prophylaxis and treatment of vascular leakage following severe trauma and shock. Mil Med Res. 2017;4:11. doi: 10.1186/s40779-017-0117-6 28361006 PMC5370457

[3] Claesson-Welsh L. Vascular permeability--the essentials. Ups J Med Sci. 2015;120(3):135–43. doi: 10.3109/03009734.2015.1064501 26220421 PMC4526869

[4] Dejana E. Endothelial cell-cell junctions: happy together. Nat Rev Mol Cell Biol. 2004;5(4):261–70. doi: 10.1038/nrm1357 15071551

[5] Sukriti S, Tauseef M, Yazbeck P, Mehta D. Mechanisms regulating endothelial permeability. Pulm Circ. 2014;4(4):535–51. doi: 10.1086/677356 25610592 PMC4278616

[6] Mai J, Virtue A, Shen J, Wang H, Yang X-F. An evolving new paradigm: endothelial cells--conditional innate immune cells. J Hematol Oncol. 2013;6:61. doi: 10.1186/1756-8722-6-61 23965413 PMC3765446

[7] Fahey E, Doyle SL. IL-1 family cytokine regulation of vascular permeability and angiogenesis. Front Immunol. 2019;10:1426. doi: 10.3389/fimmu.2019.01426 31293586 PMC6603210

[8] Xiong S, Zhang L, Richner JM, Class J, Rehman J. Interleukin-1RA mitigates SARS-CoV-2-induced inflammatory lung vascular leakage and mortality in humanized K18-hACE-2 mice. Arterioscler Thrombo Vasc Biol. 2021;41(11):2773–85. 10.1161/ATVBAHA.121.316925 PMC8545251 34496633

[9] Rabiet MJ, Plantier JL, Rival Y, Genoux Y, Lampugnani MG, Dejana E. Thrombin-induced increase in endothelial permeability is associated with changes in cell-to-cell junction organization. Arterioscler Thromb Vasc Biol. 1996;16(3):488–96. doi: 10.1161/01.atv.16.3.488 8630677

[10] Dvorak HF. Vascular permeability factor/vascular endothelial growth factor: a critical cytokine in tumor angiogenesis and a potential target for diagnosis and therapy. J Clin Oncol. 2002;20(21):4368–80. doi: 10.1200/JCO.2002.10.088 12409337

[11] Seynhaeve ALB, Vermeulen CE, Eggermont AMM, ten Hagen TLM. Cytokines and vascular permeability: an in vitro study on human endothelial cells in relation to tumor necrosis factor-alpha-primed peripheral blood mononuclear cells. Cell Biochem Biophys. 2006;44(1):157–69. doi: 10.1385/CBB:44:1:157 16456244

[12] Fajgenbaum DC, June CH. Cytokine storm. N Engl J Med. 2020;383(23):2255–73. doi: 10.1056/NEJMra2026131 33264547 PMC7727315

[13] Gu Y, Zuo X, Zhang S, Ouyang Z, Jiang S. The Mechanism behind influenza virus cytokine storm. Viruses. 2021;13(7). 10.3390/v13071362 PMC8310017 34372568

[14] Li J, Gyorffy S, Lee S, Kwok CS. Effect of recombinant human interleukin 2 on neutrophil adherence to endothelial cells in vitro. Inflammation. 1996;20(4):361–72. doi: 10.1007/BF01486739 8872500

[15] Weissmüller S, Semmler LY, Kalinke U, Christians S, Müller-Berghaus J, Waibler Z. ICOS-LICOS interaction is critically involved in TGN1412-mediated T-cell activation. Blood. 2012;119(26):6268–77. doi: 10.1182/blood-2011-12-401083 22577174

[16] Schülke S, Waibler Z, Mende M-S, Zoccatelli G, Vieths S, Toda M, et al. Fusion protein of TLR5-ligand and allergen potentiates activation and IL-10 secretion in murine myeloid DC. Mol Immunol. 2010;48(1–3):341–50. doi: 10.1016/j.molimm.2010.07.006 20965571

[17] Reinhart K, Bayer O, Brunkhorst F, Meisner M. Markers of endothelial damage in organ dysfunction and sepsis. Crit Care Med. 2002;30(5 Suppl):S302–12. doi: 10.1097/00003246-200205001-00021 12004252

[18] Fulda S, Gorman AM, Hori O, Samali A. Cellular stress responses: cell survival and cell death. Int J Cell Biol. 2010;2010:214074. doi: 10.1155/2010/214074 20182529 PMC2825543

[19] Sumpio BE, Riley JT, Dardik A. Cells in focus: endothelial cell. Int J Biochem Cell Biol. 2002;34(12):1508–12. doi: 10.1016/s1357-2725(02)00075-4 12379270

[20] Kinnunen K, Piippo N, Loukovaara S, Hytti M, Kaarniranta K, Kauppinen A. Lysosomal destabilization activates the NLRP3 inflammasome in human umbilical vein endothelial cells (HUVECs). J Cell Commun Signal. 2017;11(3):275–9. doi: 10.1007/s12079-017-0396-4 28547650 PMC5559399

[21] Kang S, Tanaka T, Inoue H, Ono C, Hashimoto S, Kioi Y, et al. IL-6 trans-signaling induces plasminogen activator inhibitor-1 from vascular endothelial cells in cytokine release syndrome. Proc Natl Acad Sci U S A. 2020;117(36):22351–6. doi: 10.1073/pnas.2010229117 32826331 PMC7486751

[22] Sun Z, Li X, Massena S, Kutschera S, Padhan N. VEGFR2 induces c-Src signaling and vascular permeability in vivo via the adaptor protein TSAd. J Exp Med. 2012;209(7):1363–77. 22689825 10.1084/jem.20111343 PMC3405501

[23] Baluna R, Vitetta ES. Vascular leak syndrome: a side effect of immunotherapy. Immunopharmacology. 1997;37(2–3):117–32. doi: 10.1016/s0162-3109(97)00041-6 9403331

[24] Jeong GH, Lee KH, Lee IR, Oh JH, Kim DW, Shin JW, et al. Incidence of capillary leak syndrome as an adverse effect of drugs in cancer patients: a systematic review and meta-analysis. J Clin Med. 2019;8(2):143. doi: 10.3390/jcm8020143 30691103 PMC6406478

[25] Xie Z, Chan E, Yin Y, Ghosh CC, Wisch L, Nelson C, et al. Inflammatory markers of the systemic capillary leak syndrome (Clarkson Disease). J Clin Cell Immunol. 2014;5:1000213. doi: 10.4172/2155-9899.1000213 25405070 PMC4232957

[26] Doukas J, Pober JS. IFN-gamma enhances endothelial activation induced by tumor necrosis factor but not IL-1. J Immunol. 1990;145(6):1727–33. 1697308

[27] Pober JS, Cotran RS. The role of endothelial cells in inflammation. Transplantation. 1990;50(4):537–44. doi: 10.1097/00007890-199010000-00001 2219269

[28] Damle NK, Doyle LV. IL-2-activated human killer lymphocytes but not their secreted products mediate increase in albumin flux across cultured endothelial monolayers. Implications for vascular leak syndrome. J Immunol. 1989;142(8):2660–9. 2522965

[29] Gogesch P, Ortega Iannazzo S, Zimmermann T, Villenave R, Sewald K, Waibler Z, et al. Analyzing IL-2-induced vascular leakage with an irAOP as tool. J Immunotoxicol. 2024;21(sup1):S79–88. doi: 10.1080/1547691X.2024.2369123 39655495

[30] Amersfoort J, Eelen G, Carmeliet P. Immunomodulation by endothelial cells - partnering up with the immune system. Nat Rev Immunol. 2022;22(9):576–88. doi: 10.1038/s41577-022-00694-4 35288707 PMC8920067

[31] Bertolino P, Schrage A, Bowen DG, Klugewitz K, Ghani S, Eulenburg K, et al. Early intrahepatic antigen-specific retention of naïve CD8+ T cells is predominantly ICAM-1/LFA-1 dependent in mice. Hepatology. 2005;42(5):1063–71. doi: 10.1002/hep.20885 16250049

[32] Stančič B, Qvarfordt B, Berglund MM, Brenden N, Sydow Bäckman M, Fransson M, et al. The blood endothelial cell chamber - an innovative system to study immune responses in drug development. Int Immunopharmacol. 2021;90:107237. doi: 10.1016/j.intimp.2020.107237 33310662

[33] de Graaf MNS, Vivas A, Kasi DG, van den Hil FE, van den Berg A. Multiplexed fluidic circuit board for controlled perfusion of 3D blood vessels-on-a-chip. Lab Chip. 2022;23(1):168–81. 36484766 10.1039/d2lc00686c PMC9764810

[34] Lidington EA, McCormack AM, Yacoub MH, Rose ML. The effects of monocytes on the transendothelial migration of T lymphocytes. Immunology. 1998;94(2):221–7. doi: 10.1046/j.1365-2567.1998.00473.x 9741344 PMC1364208

[35] Claser C, Nguee SYT, Balachander A, Wu Howland S, Becht E, Gunasegaran B, et al. Lung endothelial cell antigen cross-presentation to CD8+T cells drives malaria-associated lung injury. Nat Commun. 2019;10(1):4241. doi: 10.1038/s41467-019-12017-8 31534124 PMC6751193

