# Supplementary material for: Immune cells play a critical role in cytokine- and endotoxin-mediated endothelial permeability
Source: PLoS One. 2025 Aug 14;20(8):e0329700. doi: 10.1371/journal.pone.0329700 (PMC12352842; doi:10.1371/journal.pone.0329700)
Supplement: S1 Fig — Data for ICAM-1, VCAM-1, and E-Selectin (Fig 1) and IL-6, IL-8, and MCP-1 (Fig 3) were analyzed using Spearman correlation pairing all combinations with the same underlying conditions. (A) Spearman correlation results pairing ICAM-1, VCAM-1, and E-selectin. (B) Spearman correlation results pairing of IL-6, IL-8, and MCP-1. (C) Spearman correlation results pairing ICAM-1, VCAM-1, and E-selectin with IL-6, IL-8, and MCP-1. The correlation coefficient (r) was defined as: r < 0.5 low positive correlation; r < 0.7 moderate positive correlation; r < 0.9 high positive correlation. (PDF) [file pone.0329700.s001.pdf]

Supplemental Fig. S1

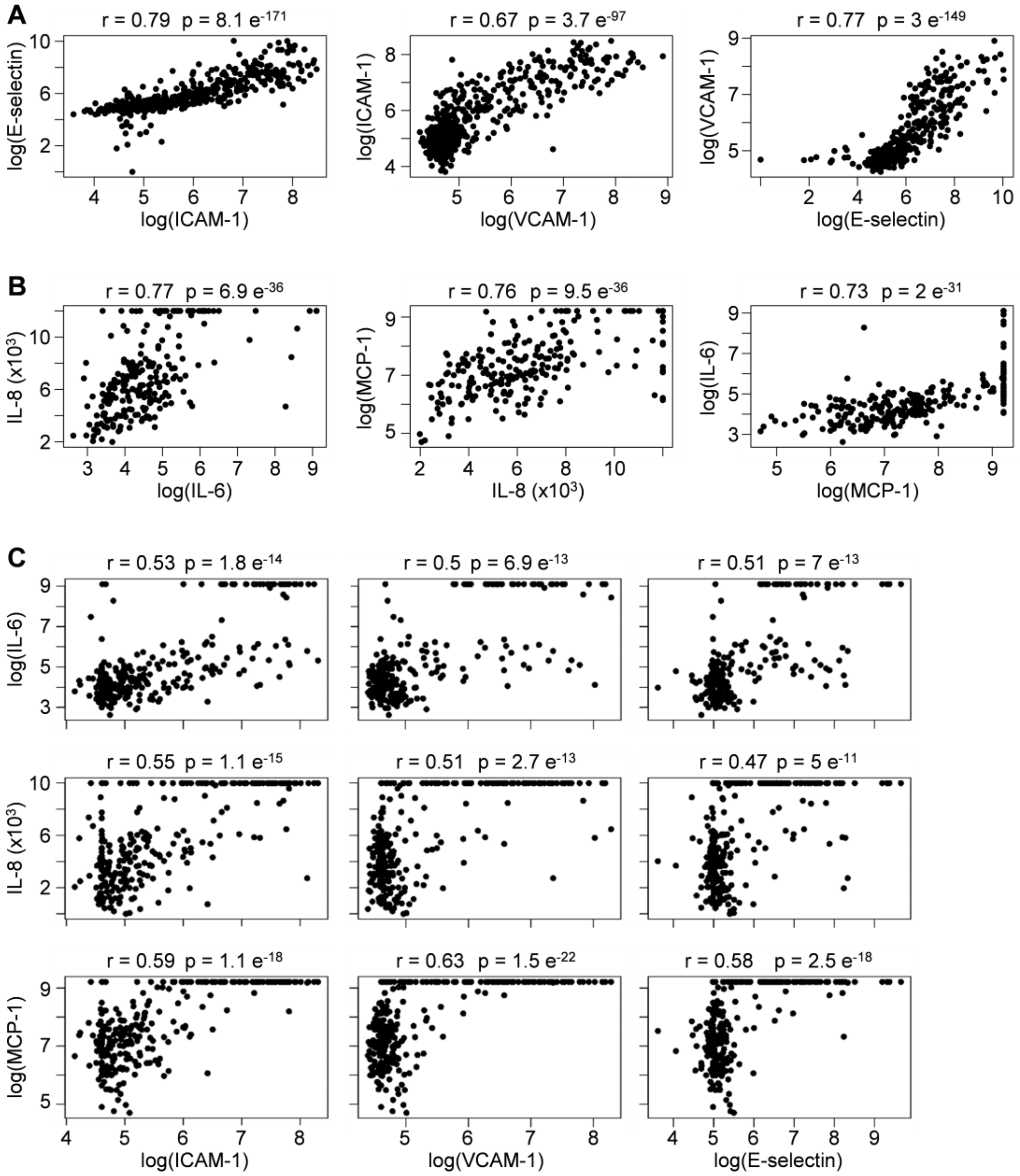

Supplemental Fig. S1. Expression of ICAM-1, VCAM-1, E-Selectin, IL6, IL-8, and MCP-1 show a positive correlation. Data for ICAM-1, VCAM-1, and E-Selectin (figure 1) and IL-6, IL-8, and MCP-1 (figure 3) were analyzed with spearman correlation pairing all combinations with the same underlying conditions. **(A)** Spearman correlation results pairing ICAM-1, VCAM-1, and E-selectin. **(B)** Spearman correlation results pairing of IL-6, IL-8, and MCP-1. **(C)** Spearman correlation results pairing ICAM-1, VCAM-1, and E-selectin with IL-6, IL-8, and MCP-1. The correlation coefficient (r) was defined as: $r < 0.5$ low positive correlation; $r < 0.7$ moderate positive correlation; $r < 0.9$ high positive correlation.
